# Predictive biomarkers for immune-related adverse events in cancer patients treated with immune-checkpoint inhibitors

**DOI:** 10.1186/s12865-024-00599-y

**Published:** 2024-01-24

**Authors:** Jingting Wang, Yan Ma, Haishan Lin, Jing Wang, Bangwei Cao

**Affiliations:** 1 Department of Oncology, Beijing Friendship Hospital, Capital Medical University, #95 Yong An Road, Xicheng District, Beijing, China; 2 Radiotherapy Department, Shijingshan Teaching Hospital of Capital Medical University Beijing, #24 Shijingshan Road, Shijingshan District, Beijing, 100040 China

**Keywords:** Immunotherapy, Immune-related adverse events, Predictive biomarker, Real-world retrospective study

## Abstract

**Purpose:**

The objective of this study was to identify potential predictors of immune-related adverse events (irAEs) in cancer patients receiving immune checkpoint inhibitor therapy among serum indexes, case data, and liquid biopsy results.

**Methods:**

We retrospectively analyzed 418 patients treated with anti-programmed cell death 1(PD-1)/PD-1 ligand (PD-L1) inhibitors from January 2018 to May 2022 in our cancer center. We identified factors that correlated with the occurrence of irAEs and evaluated associations between irAEs and anti-PD-1/PD-L1 inhibitor responses.

**Results:**

The incidence of irAEs was 42.1%, and pneumonitis (9.1%), thyroid toxicity (9.1%), cardiotoxicity (8.1%), and dermatologic toxicity (6.9%) were the four most common irAEs. Multivariate logistic analysis identified female sex, antibiotic use, higher post-treatment neutrophil-to-lymphocyte ratio (NLR), and higher baseline circulating tumor cell (CTC) level, as predictive biomarkers for the occurrence of irAEs. A lower baseline prognostic nutritional index (PNI), body mass index (BMI) ≥ 25 kg/m^2^, and higher post-treatment lactate dehydrogenase (LDH) level were predictive factors for more severe irAEs (higher severity grade). Patients without irAEs had better overall survival than those with irAEs. Specifically, pneumonitis and cardiotoxicity were found to be significant predictors of poor prognosis in the irAE subgroup with different organ-related irAEs. Low-dose steroid (dexamethasone 10 mg) treatment had no significant effect on outcomes.

**Conclusions:**

Gender, antibiotic use, post-treatment NLR, and baseline CTC level are potential predictive biomarkers of irAEs, while baseline PNI, BMI, and post-treatment LDH may predict the severity of irAEs. The predictive effect of irAE occurrence on survival benefit may depend on the type of irAE.

**Supplementary Information:**

The online version contains supplementary material available at 10.1186/s12865-024-00599-y.

## Introduction

**Fig. 1:**
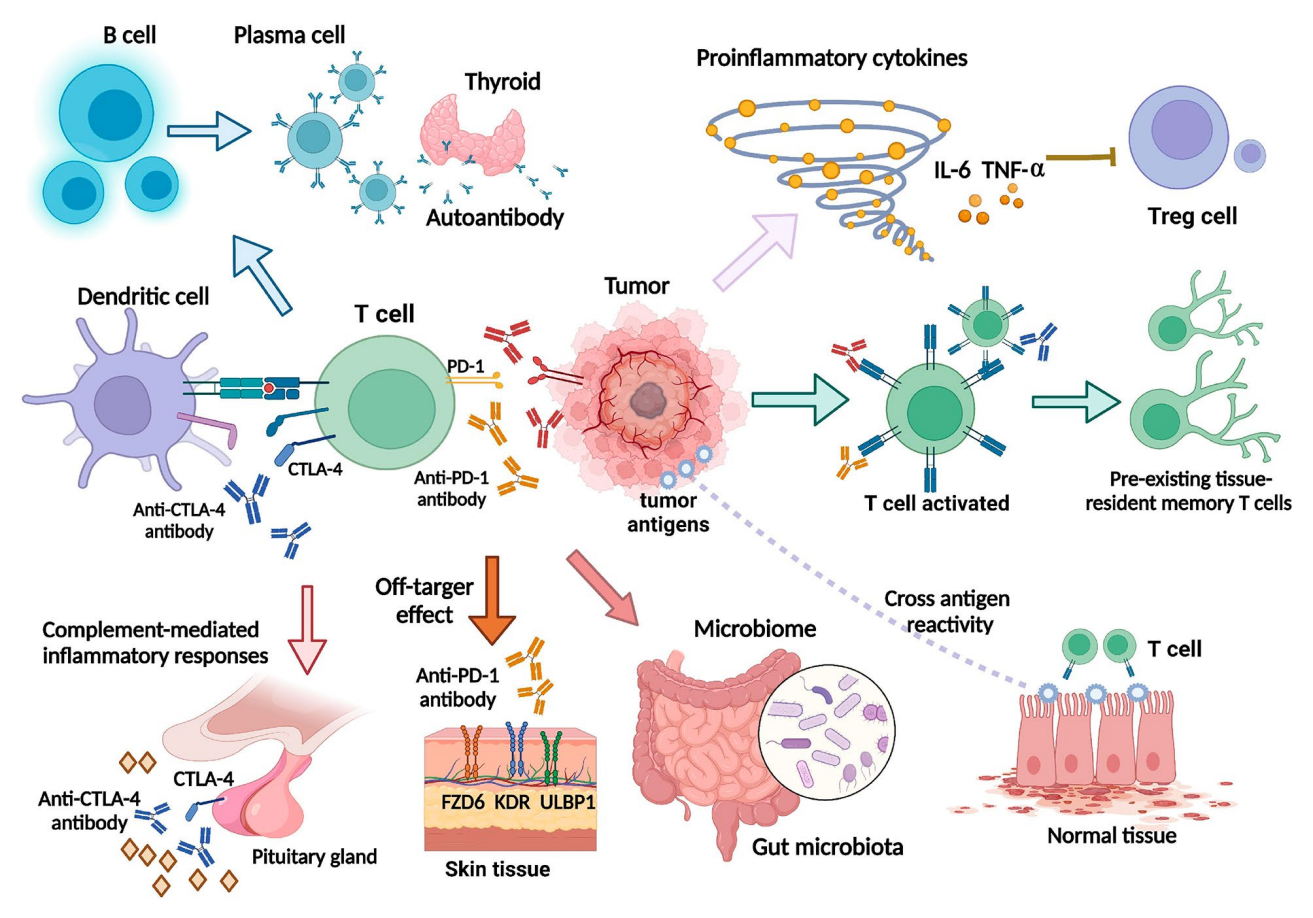
Mechanisms of irAE development

Immune checkpoint inhibitor (ICI) therapy has shown significant efficacy in a variety of malignant tumors and changed the treatment landscape for advanced forms of many cancers [1]. ICIs activate T cells to enhance immunity and improve the killing effect on tumor cells with less immunosuppression than traditional chemotherapies [2]. However, the process of immunotherapy causes disruption of immunity balance, which can lead to immune-related adverse events (irAEs). Potential mechanisms for the development of irAEs are illustrated in Fig. 1 and include increased initiation and activation of antigen-specific T cells, leading to the formation of autoreactive T cells, which attack both malignant and normal tissues; the cross immune response; increases in B cell clonality and autoantibodies; increased production of pro-inflammatory cytokines, such as interleukin 6; activation of preexisting tissue-resident memory T cells; enrichment of certain gut microbiota species that can induce inflammatory response syndromes; enhanced complement-mediated inflammatory response to direct binding antibody against cytotoxic T lymphocyte antigen 4 expression in normal tissues; and off-target effects of anti-programmed cell death 1(PD-1) inhibitors [3–6]. With the increasing application of immunotherapy in cancer treatment and a clearer understanding of the mechanism of irAEs, great progress has been made in the monitoring, prevention, and treatment of irAEs.

IrAEs can occur in any organ system, and different types of tumor-specific immune microenvironments may induce specific irAEs [7]. In addition, different immunotherapy drugs induce irAEs in different organs, such as pneumonitis and hepatitis observed with Pembrolizumab, endocrine toxicity with Nivolumab, hypothyroidism with Atezolizumab, and dermatologic toxicity with Camrelizumab. However, once grade 3/4 irAEs occur, immunotherapy should be stopped immediately and high-dose glucocorticoids and/or immunosuppressants administered according to the patient’s situation [4].

Early assessment and identification of irAEs is important for maximizing the immune anti-tumor effect of ICI treatment to improve patient outcomes while preventing negative outcomes associated with irAEs. Thus, accurate predictive biomarkers for irAEs are needed. In addition, the correlations between the occurrence of irAEs and clinical outcomes and ICI benefits need to be well understood. In the present study, we aimed to identify potential predictors of irAEs by investigating related factors such as peripheral blood biomarkers, medical records, and liquid biopsy results in real-world cancer patients receiving anti-PD-1/PD-1 ligand (PD-L1) therapy, and to evaluate the relationship between irAEs and the clinical efficacy of anti-PD-1/PD-L1 inhibitors.

## Materials and methods

### Study design and patient population

This retrospective study included patients with malignant tumors treated with an anti-PD-1/PD-L1 inhibitor during hospitalization at the Beijing Friendship Hospital Cancer Center, Capital Medical University between January 1, 2018 and May 1, 2022. All malignancies were diagnosed by pathological evaluation. All patients had overall complete case data for assessment of treatment efficacy, disease progression, or treatment failure as well as data relating to irAEs. The follow-up period began with initiation of anti-PD-1/PD-L1 inhibitor therapy and ended with disease progression, confirmed death, or loss to follow-up. The following exclusion criteria were used: past medical history or test results indicating the presence of a definitely inherited disease; presence of severe autoimmune disease or other diseases, such as cardiovascular, lung, kidney, or other organ diseases; and absence of important results from medical records, such as serological data from before or after treatment. The anti-PD-1/PD-L1 inhibitors used for treatment in this study mainly included Nivolumab, Pembrolizumab, Atezolizumab, Durvalumab, Sintilimab, and Camrelizumab. The study protocol was approved by the Institutional Review Board of Beijing Friendship Hospital (2020-P2-176-01).

### Data collection

Clinical and pathological data for all patients treated with anti-PD-1/PD-L1 inhibitors were collected by consulting electronic medical records and included age, sex, body mass index (BMI), Eastern Cooperative Oncology Group performance status (ECOG PS), medical records regarding other chronic diseases (e.g., cardiovascular diseases, diabetes, liver and kidney diseases, autoimmune diseases, etc.), tumor type, pathological type, common mutated gene, circulating tumor cell (CTC) level, and peripheral blood test results. The recorded clinical data also included Tumor Node Metastasis (TNM) staging, treatment details, use of antibiotics (defined as treatment with antibiotics 3 months before or 1 month after PD-1/PD-L1 inhibitor treatment), steroid use, and treatment efficacy.

The relationship between the administered ICI and the resulting irAEs was reported by each patient’s physician in their medical files and systematically evaluated by members of the pharmacovigilance team. Safety was evaluated based on the incidence rate of any-grade irAEs and that of grade 3 or 4 irAEs according to the National Cancer Institute’s Common Terminology Criteria for Adverse Events, version 4.0. Any potential adverse events with an immune basis requiring close monitoring and/or possible intervention with hormonal or immunosuppressive therapy were considered irAEs. Confirmation of irAEs involved both the patient and the attending physician through analysis of medical records and follow-up interviews.

In this study, specific AEs were considered irAEs, including pneumonia, diarrhea or colitis, hepatitis, nephritis, cardiotoxicity (myocarditis, heart failure, and myocardial infarction), dermatologic toxicity, hyponatremia, oral mucositis, encephalitis, myathenia gravis, hemolysis, and endocrine AEs, that occurred during the treatment period or within 100 days of the last study treatment. Endocrine AEs, including adrenal insufficiency, hypopituitarism, hypothyroidism, thyroiditis or hyperthyroidism, and diabetes mellitus also were considered irAEs.

Treatment efficacy was assessed in terms of overall survival (OS), which was recorded from the beginning of treatment until the observation of death from any cause during follow-up. Peripheral blood tests included routine blood cell counts (absolute neutrophil count, absolute lymphocyte count, platelet count, absolute eosinophil count [AEC], etc.), biochemical measurements (protein concentration, indicators of liver and kidney function, myocardial enzymes, electrolytes, lactate dehydrogenase, lipid levels, etc.), coagulation tests, measures of thyroid function and auto-antibodies, tumor biomarkers, T and B lymphocyte subsets, and immunoglobulins. Only measurements taken within 3 days prior to administration of the first cycle of anti-PD-1/PD-L1 inhibitor therapy were used as baseline measurements. Post-treatment measurements were taken on day 14 after the first cycle of anti-PD-1/PD-L1 inhibitor treatment.

### Statistical analysis

All data were statistically analyzed using SPSS Statistics, version 22.0. Forest plots and survival curves were prepared with GraphPad Prism 9, version 9.4.1. Illustrations were made in BioRender. Receiver operating characteristic (ROC) curve analysis was performed to evaluate the performance of potential predictive markers and to determine the optimal cut-off values for the biomarkers. Survival curves were estimated by the Kaplan–Meier method, and the survival times of different groups were then analyzed using the log-rank test. Associations of peripheral blood markers and other biomarkers with irAEs were evaluated by univariate and multivariate logistic regression analyses. Biomarkers for irAEs of different grades were identified by multivariate logistic regression. The threshold for statistical significance was set at *P* < 0.05 in all tests.

## Results

### Clinical characteristics of patients and irAEs

**Fig. 2:**
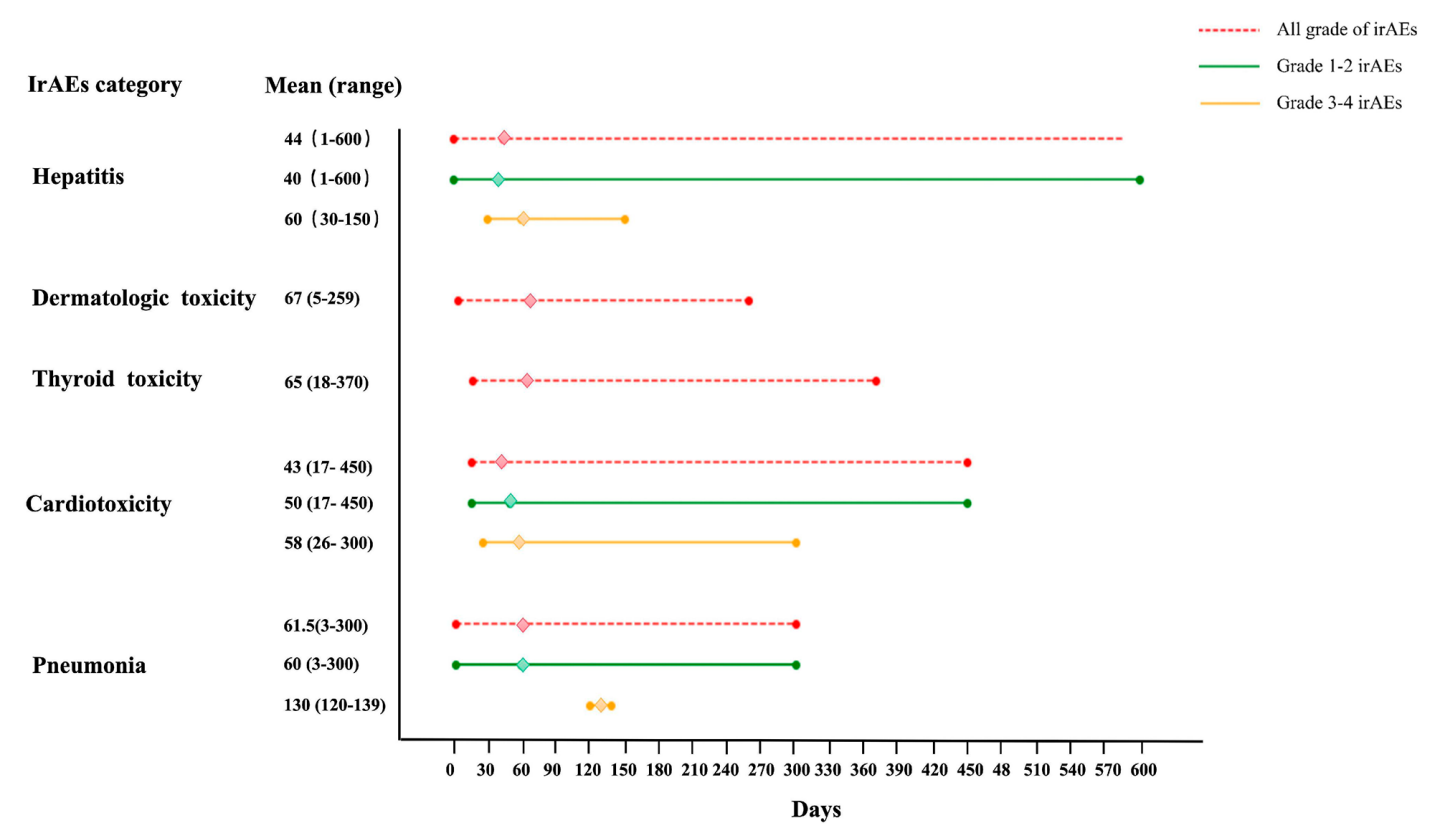
Occurrence time profiles for different types of irAEs and different grades of irAEs

A total of 418 patients with a solid tumor received anti-PD-1/PD-L1 inhibitor treatment during the study period and were included in the present study. The patient characteristics are summarized in Table 1. The male/female ratio was 2.48, and the median age at the start of ICI treatment was 64.1 years (range, 21–87 years). Overall, 50 (12%) of the 418 patients received PD-1/PD-L1 monotherapy and 282 (67.5%) patients received a combination of PD-1/PD-L1 inhibitors and chemotherapy. Another 54 (12.9%) patients received PD-1/PD-L1 inhibitors combined with targeted therapy. The main tumor types were lung cancer, head and neck cancers, and digestive system cancers. At the time ICI initiation, 280 (67%) patients had been diagnosed with stage IV cancer, and 108 (25.8%) patients had been diagnosed with stage III cancer. Most patients (384/418, 91.9%) had an ECOG PS of 0 or 1 and 296 (70.8%) patients had a BMI in the range of 18.5–24.9 kg/m^2^. Among the 418 patients, irAEs occurred in 176 patients (42.1%). These included grade 1 irAEs in 87 (49.4%) patients, grade 2 irAEs in 75 (42.6%) patients, grade 3 irAEs in 7 (4.0%) patients, and grade 4 irAEs in 7 (4.0%) patients. Pneumonia (9.1%), thyroid toxicity (9.1%), cardiovascular toxicity (8.1%), and dermatologic toxicity (6.9%) were the four most common irAEs in this study (Table 2). We reviewed the occurrence times of pneumonia, cardiotoxicity, thyroid toxicity, dermatologic toxicity, and hepatitis, and the specific time profiles for the occurrence of different types of irAEs as well as irAEs of different grades after the first treatment are presented in Fig. 2. Most irAEs occurred within 70 days. Cardiotoxicity appeared early related to other irAEs, and among severe irAEs (grades 3–4), both cardiotoxicity and hepatitis appeared earlier. Figure 2 also shows how severe irAEs tended to occur later than milder irAEs within the same irAE category. Comparison of the occurrence times of common irAEs suggests that the most important time window for irAE monitoring is within the first 70 days after immunotherapy, with dynamic monitoring also required during follow-up. Moreover, irAEs of different organs and grades should be monitored within the respective relevant time periods.

**Table 1:** Characteristics of cancer patients treated with anti-PD-1/PD-L1 inhibitors

Patient characteristics	Patients treated with anti-PD-1/PD-L1 therapy (*n* = 418), n (%)
**Gender**	
Male	298(71.3)
Female	120(28.7)
**Age at initiation of anti-PD-1/PD-L1 therapy (years)**	
Median	64.1
Range	21–87
**Tumor type**	
Lung cancer	131(31.3)
Head and neck cancer	57(13.6)
Esophageal carcinoma	51(12.2)
Gastric carcinoma	51(12.2)
Urothelial carcinoma	28(6.7)
Colorectal cancer	23(5.5)
Reproductive system cancer	19(4.5)
Liver cancer	17(4.1)
Gallbladder carcinoma and bile duct carcinoma	13(3.1)
melanoma	8(1.9)
Others	20(4.8)
**TNM clinical classification**	
III	108(25.8)
IV	280(67.0)
Unknown	30(7.2)
**BMI**	
18.5–24.9	296(70.8)
25-29.9	122(29.2)
**ECOG score standard**	
0	94(22.5)
1	290(69.4)
2	26(6.2)
3	8(1.9)
**Treatment**	
Immunotherapy	50(12.0)
Immunotherapy + chemotherapy	282(67.5)
Immunotherapy + targeted therapy	54(12.9)
Immunotherapy + chemotherapy + targeted therapy	32(7.6)
**Baseline CTC**	
Median	14.8
Range	1.8–86.5
**The occurrence of irAEs**	
irAEs	176(42.1)
No-irAEs	242(57.9)

**Table 2:** Details of irAEs that occurred among the patients

Grade of irAEs	Number of patients (%)
Grade 1	87 (20.8%)
Grade 2	75 (17.9%)
Grade 3	7 (1.7%)
Grade 4	7 (1.7%)
**IrAEs category**	
Pneumonia	38 (9.1%)
Thyroiditis, hypothyroidism, hyperthyroidism	38 (9.1%)
Cardiovascular toxicity	34 (8.1%)
Dermatologic toxicity	29 (6.9%)
Hepatitis	17 (4.1%)
Diarrhea, colitis	5 (1.2%)
Hyponatremia	3 (0.7%)
Oral mucositis	2 (0.5%)
Adrenal insufficiency	2 (0.5%)
Nephritis	2 (0.5%)
Diabetes mellitus	2 (0.5%)
Encephalitis	1 (0.2%)
Myathenia gravis	1 (0.2%)
Hemolysis	1 (0.2%)
Hypopituitarism	1 (0.2%)
**Total patients irAEs**	**176 (42.1%)**

### Predictive biomarkers for irAEs

**Fig. 3:**
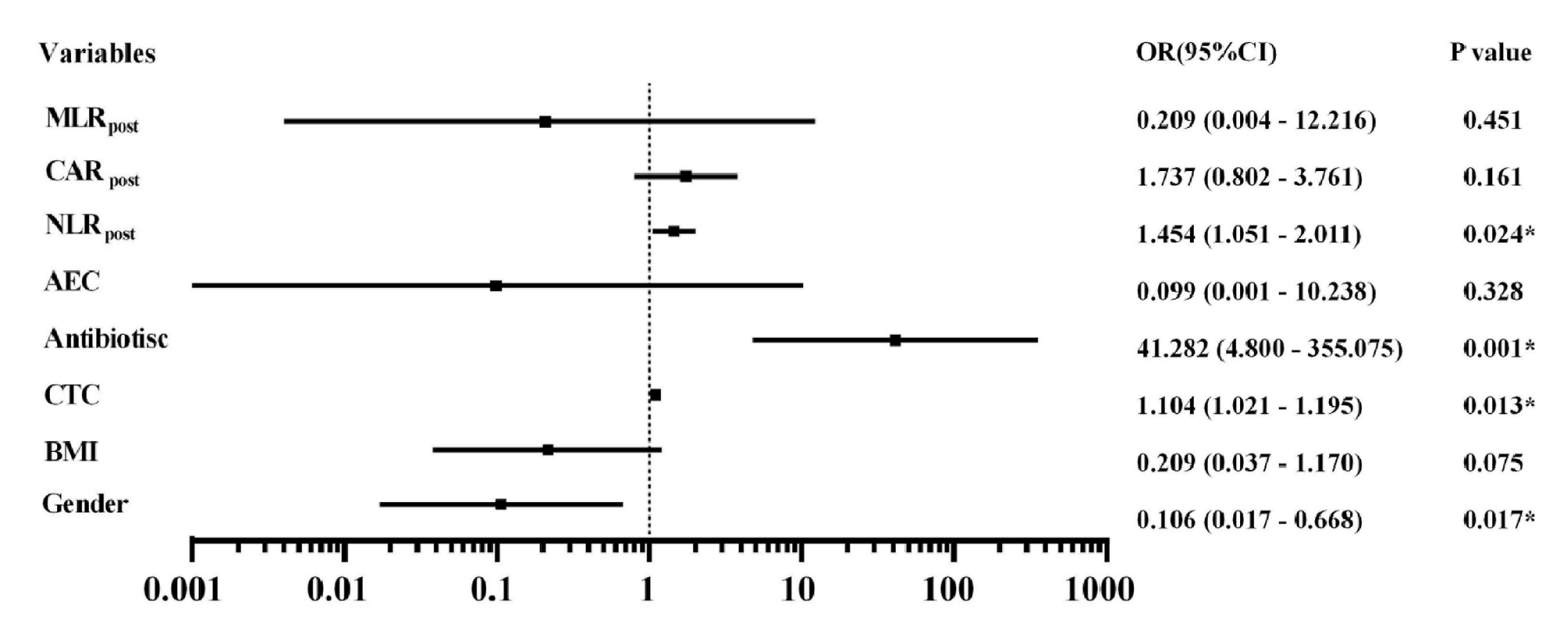
Forest plot of multivariate logistic regression results for biomarkers of irAEs

To identify factors related to the occurrence of irAEs, univariate logistic regression analysis was performed to detect significant associations between clinical variables and irAEs. The univariate logistic regression analysis showed that BMI at baseline (*P* = 0.02, odds ratio [OR]: 0.492, 95% confidence interval [CI] 0.314–0.770), baseline CTC level (*P* = 0.09, OR: 1.090, 95% CI 1.022–1.163), antibiotic use (*P* < 0.001, OR: 6.152, 95% CI 3.690–10.256), baseline AEC (*P* = 0.003, OR: 10.245, 95% CI 2.221–47.256), post-treatment neutrophil to lymphocyte ratio (NLR) (*P* = 0.01, OR: 1.129, 95% CI 1.015–1.213), post-treatment C-reactive protein to albumin ratio (CAR) (*P* < 0.001, OR: 1.842, 95% CI 1.428–2.375), and post-treatment monocyte to lymphocyte ratio (MLR) (*P* = 0.01, OR: 3.246, 95% CI 1.323–7.965) were significantly associated with the occurrence of irAEs. Multivariate logistic analysis was performed for the factors found to be significant on univariate analysis and gender (Fig. 3). From this analysis, gender (*P* = 0.017, OR: 0.106, 95% CI 0.017–0.668), antibiotic use (*P* = 0.001, OR: 41.282, 95% CI 4.800–355.075), a high post-treatment NLR (*P* = 0.024, OR: 1.454, 95% CI 1.051–2.011), and a high baseline CTC level (*P* = 0.013, OR: 1.104, 95% CI 1.021–1.195) were identified as independent predictive biomarkers for irAEs. Compared with male patients, female patients have an increased risk of irAEs.

### Performance of peripheral blood variables as predictive biomarkers for irAEs

**Fig. 4:**
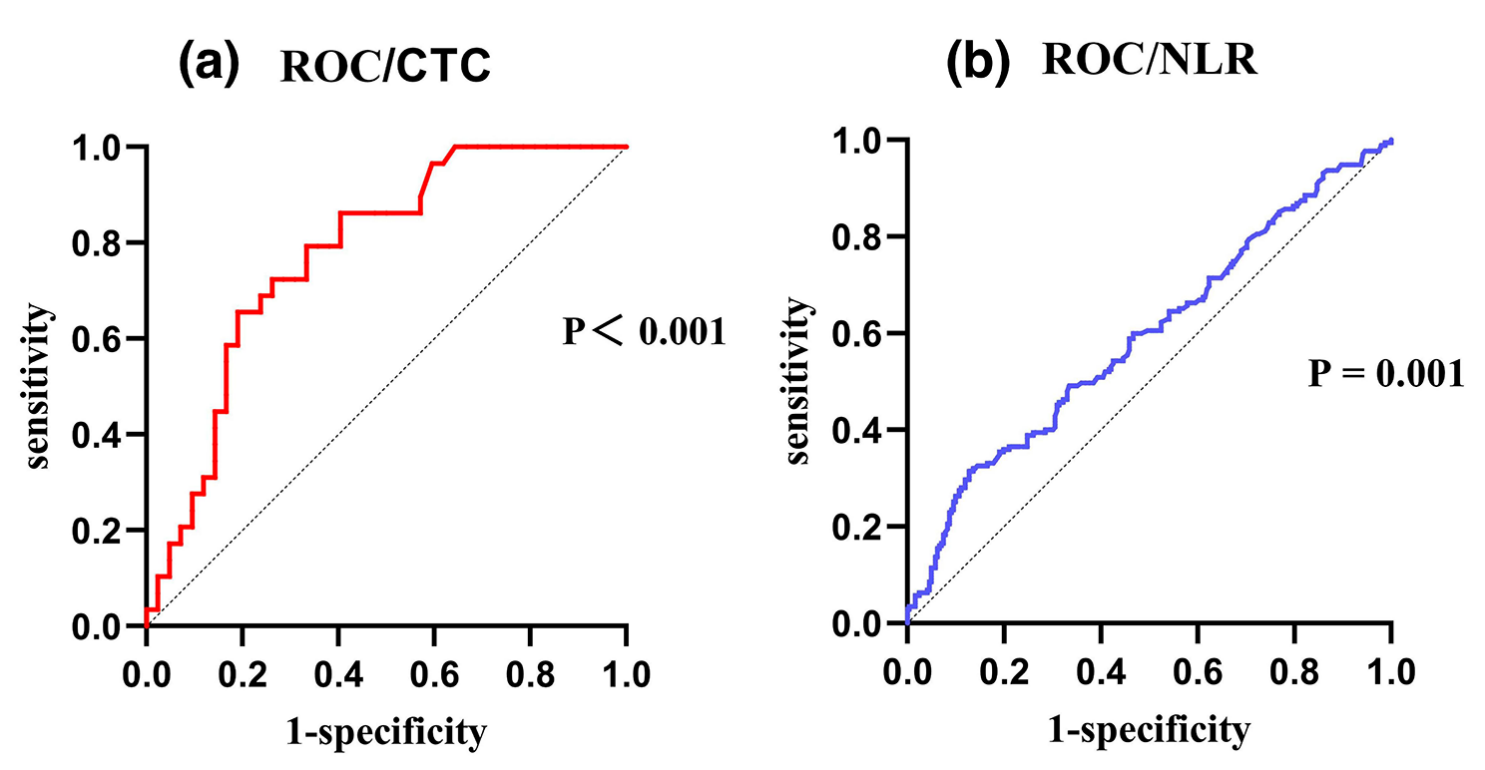
ROC curves for the ability of post-treatment NLR **(a)** and baseline CTC **(b)** to predict irAEs

Using irAEs as the result variable, we generated ROC curves for post-treatment NLR and the baseline CTC level (Fig. 4). From this analysis, the optimal cut-off value for the baseline CTC level was 15.0 FU/ml, with an area under the curve (AUC) value of 78%, sensitivity of 66%, specificity of 81%, and Youden index of 0.47. The optimal cut-off value for the post-treatment NLR was 4.5, with an AUC value of 60%, sensitivity of 31%, specificity of 87%, and Youden index of 0.19. We then compared the incidence rates of irAEs among groups of patients separated by these cut-off values (Table 3). The incidence of irAEs was significantly higher in the high-CTC group (53.3%) than in the low-CTC group (31.7%; *P* < 0.001), and the incidence of irAEs was lower in the low post-treatment NLR group (36.3%) than in the high post-treatment NLR group (64.4%; *P* = 0.010).

**Table 3:** Associations between peripheral blood markers and irAEs.

Blood parameter	Cut-off value	irAEs, *n* (%)	*P* value
NLR _post_	4.5		0.001*
Low(*n*= 331)		120/331 (36.3%)	
High(*n* = 87)		56/87 (64.4%)	
CTC	15.0 FU/ml		<0.001*
Low(*n*= 41)		13/41 (31.7%)	
High(*n* = 30)		16/30 (53.3%)	

### Predictive biomarkers for irAE severity

The irAEs were divided into subgroups according to the different grades of irAEs (grades 1–4) to explore the predictive performance of different clinical variables for the severity of irAEs (Supplement 1). We found that a baseline BMI < 25 kg/m^2^ was a protective factor (*P* = 0.021); that is, the severity grade of irAEs among patients with BMI < 25 kg/m^2^ was lower than that among those with BMI ≥ 25 kg/m^2^. In addition, among the peripheral blood factors, the severity grades of irAEs were higher in patients with a lower baseline prognostic nutritional index (PNI) (*P* = 0.048) and post-treatment higher LDH level (*P* = 0.031).

### Associations between irAEs and OS after ICI therapy

**Fig. 5:**
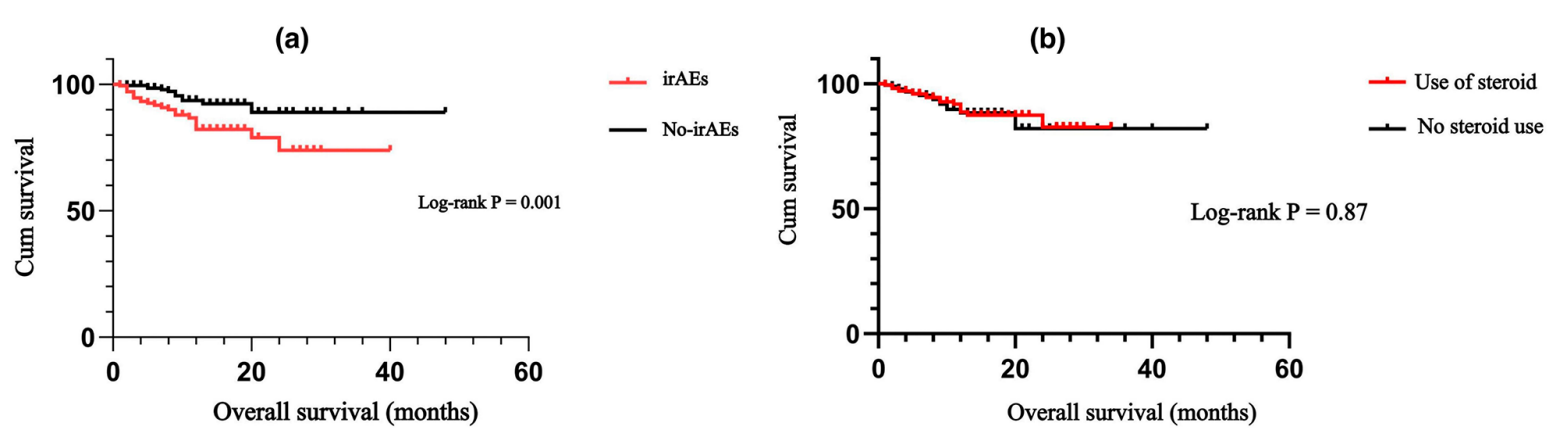
Kaplan–Meier survival curves for the association of irAE occurrence (a) and steroid therapy (b) with OS

**Fig. 6:**
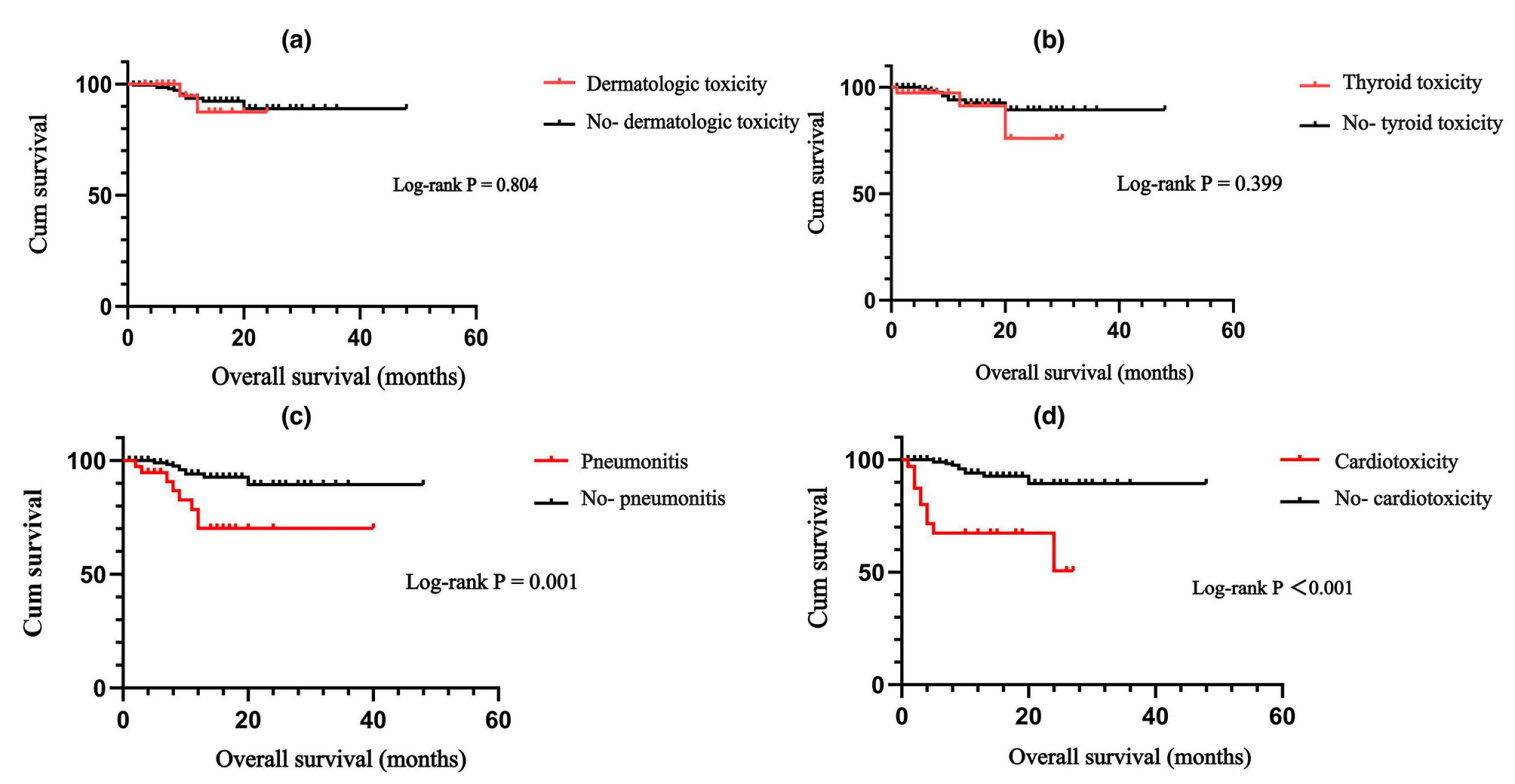
Kaplan–Meier survival curves for the association of dermatologic toxicity **(a)**, thyroid toxicity **(b)**, pneumonitis **(c)**, and cardiotoxicity **(d)** with OS

Kaplan–Meier survival curves were generated to explore the impact of the presence or absence of irAEs on OS. The results demonstrated that patients who did not experience irAEs had better OS than patients who did experience irAEs (*P* = 0.001; Fig. 5). We examined the associations between the four common irAEs (i.e., dermatologic toxicity, thyroid toxicity, pneumonitis, and cardiotoxicity) and OS (Fig. 6). Among the vital organ-related irAEs, patients with cardiotoxicity showed extremely poor OS compared with patients without cardiotoxicity (*P* < 0.001). Patients who developed pneumonia during immunotherapy also had a shorter OS than those who did not (*P* = 0.001). OS did not differ significantly between patients who did or did not develop thyroid toxicity (*P* = 0.399) or between patients who did or did not experience dermatologic toxicity (*P* = 0.804).

### Association between steroid treatment during ICI therapy and OS

Among the 418 patients included in this study, 220 patients received low-dose steroid treatment (dexamethasone 10 mg) during ICI therapy, and 198 did not. Kaplan–Meier curve analysis of the effect of steroid therapy on patients’ OS showed treatment with low-dose steroids had no significant effect on OS (*P* = 0.87; Fig. 5).

## Discussion

**Fig. 7:**
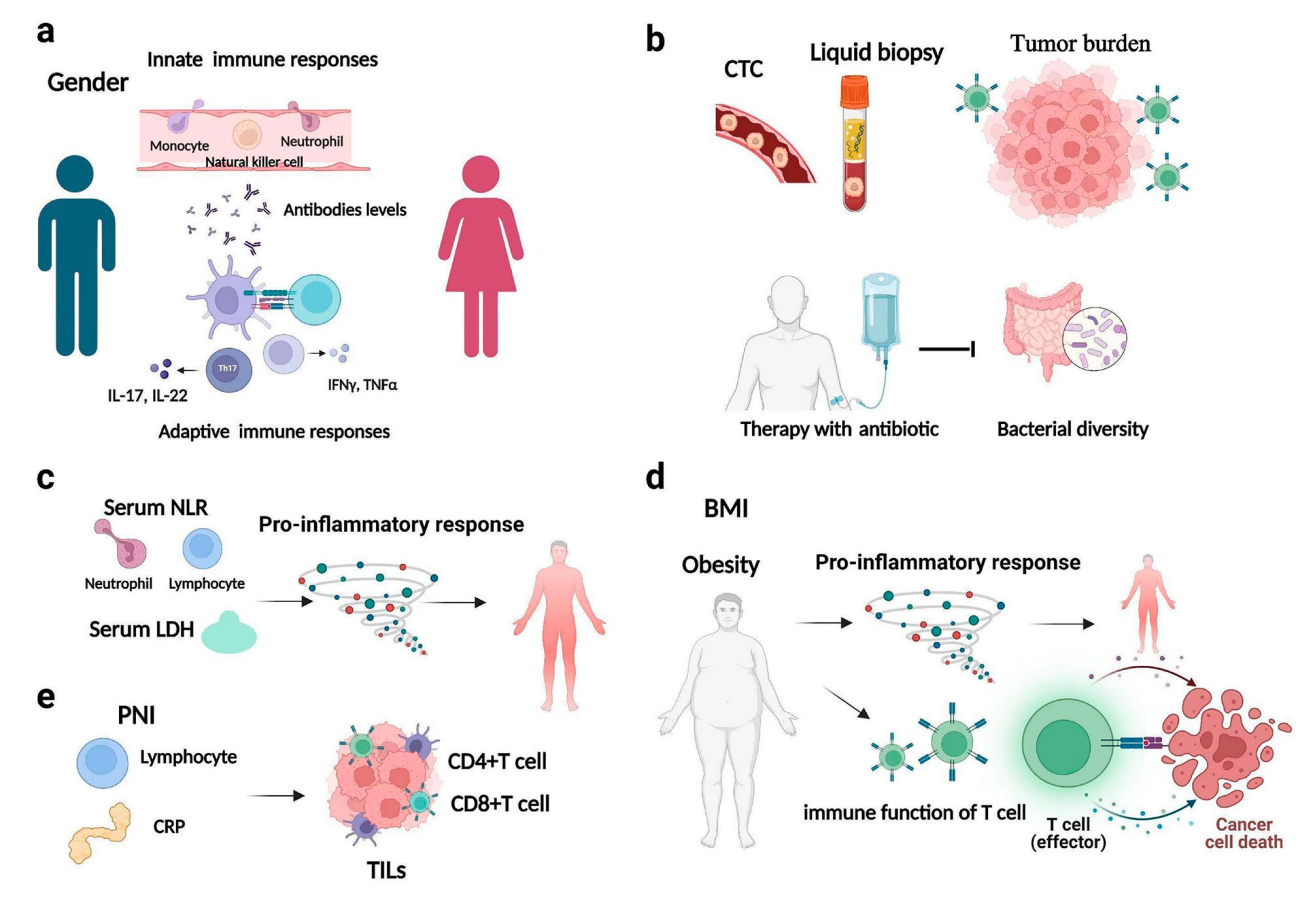
Potential predictive biomarkers and the corresponding underlying mechanisms for irAEs

ICI therapy has demonstrated long-lasting therapeutic effects for a variety of tumor types. However, irAEs may result from disruption of the balance of the normal immune system during immunotherapy. The occurrence of irAEs can damage various systems throughout the whole body, and treatment of irAEs may require interruption of immunotherapy or use of steroids, which will weaken the effect of immunotherapy. In addition, severe irAEs can directly cause a fatal immune inflammatory storm, resulting in multiple organ damage and death. Therefore, early prediction and identification of irAEs is the key to the whole-course management of immunotherapy. Our present study explored potential biomarkers for irAEs, and the results provide insight for inferring potential underlying mechanisms (Fig. 7).

This retrospective study found that female sex, antibiotic user, a higher post-treatment NLR, and a higher baseline CTC were predictive biomarkers for the occurrence of irAEs in patients treated with IC therapy. Gender is known to affect some aspects of the human immune response to external and autoantigens. A previous pan-cancer analysis identified gender-specific characteristics in the tumor microenvironment (TME) and showed that gender has an impact on tumor mutation burden (TMB), immune cell counts, immune checkpoint genes, and related functional pathways in the TME [8]. Due to the complex interaction between hormones, genes, behavior, and microbiome, women have higher innate and adaptive immune responses than men as well as increased susceptibility to autoimmune diseases, resulting in a higher risk of irAEs [9]. The study by Unger et al., which explored gender differences in the responses to therapies such as immunotherapy, assessed the differences in the risk of serious irAEs between women and men across treatment modalities and found that among those receiving immunotherapy, women had a 49% greater risk of irAEs than men, and the grade of irAEs was higher among women [10]. However, the relatively small numbers of women enrolled in large randomized controlled trials of immunotherapy and in the present study may have biased the results. Further studies to establish associations between sex hormones and irAEs as well as sex differences in the occurrence of irAEs are needed to better understand the underlying mechanisms and identify therapeutic targets to improve treatment safety in patients of different genders.

Immunomodulatory cytokines and systemic inflammatory markers are known to be important factors in cancer development and response to immunotherapy. Certain routine test results derived from peripheral blood samples, such as the NLR, platelet-to-lymphocyte ratio and CAR, have been considered indicators of systemic inflammation in recent years and are correlated with classical inflammatory mediators (IL-6, IL-8, IL-10, etc.). However, compared with classical inflammatory mediators, these peripheral blood variables are more widely available in clinical practice, do not increase the cost of examination for patients, and have higher stability [11]. In the present study, patients with higher post-treatment NLR were more likely to develop irAEs. This may be because the NLR reflects the balance between the tumor inflammatory response and anti-tumor immunity. Neutrophils extensively infiltrate the TME, and their excessive increase can result in the secretion of a variety of tumor-promoting substances to facilitate the formation of the TME and promote the immune response. In addition, studies have shown that a higher NLR is significantly associated with poorer clinical benefit from immunotherapy [12]. However, in the present study, the reliability of the post-treatment NLR for predicting irAEs was questionable, with an AUC value of 60% and a sensitivity of only 31%. The occurrence of irAEs was significantly associated with a lower baseline NLR in a previous study, and these correlations need to be further explored [13]. Overall, these results suggest that clinicians should pay attention to the NLR and other immune-inflammatory factors in the evaluation of the safety and efficacy of immunotherapy.

The CTC level is one of the most important results of liquid biopsy, as it represents the number of tumor cells that are circulating in the peripheral blood after being shed from a primary or metastatic tumor [14]. CTC detection can capture and detect the measurable presence of tumor cells in peripheral blood to monitor the changing trend in the CTC level, in order to monitor tumor dynamics in real time and evaluate the immunotherapy response [15]. In the present study, the baseline CTC level of patients was found to predict the occurrence irAEs with high sensitivity and specificity, as well as reliable predictive ability. Castello et al. explored the CTC level and metabolic parameters in non-small cell lung cancer (NSCLC) patients treated with ICI therapy and found that an elevated CTC count was an important prognostic and predictive factor together with metabolic tumor burden, suggesting that large and high metabolic cancers may have the potential to shed large numbers of CTCs into the bloodstream, increasing the likelihood of distant metastasis [16]. Previous studies have shown a significant positive correlation between the incidence of irAEs and the corresponding TMB in multiple cancer types, suggesting that potential neoantigens caused by a high TMB may be responsible for increasing the risk of irAEs [17]. Patients with a higher CTC level have a higher tumor burden, making them more prone to irAEs, worsening the immunotherapy effect, and leading to a poor prognosis [18]. Together these results suggest the important role of monitoring the CTC level during immunotherapy in clinical practice.

In the present study, antibiotic use within 3 months before or 1 month after PD-1/PD-L1 inhibitor therapy was a significant independent predictor of irAEs. Previous studies have reported an association between antibiotic use and a higher risk of diarrhea and moderate-to-severe immune-mediated colitis in ICI patients, suggesting a potential impact of antibiotics on ICI-induced colitis through the microbiome immune axis [19]. Jing et al. [20] comprehensively demonstrated the correlation between the occurrence of irAEs and antibiotics, and found an inverse relationship between microbial diversity and irAE-related factors/pathways, of which significantly related genes were mainly enriched in the neutrophil activation and T-cell activation pathways. Therefore, decreased microbial diversity caused by antibiotic use may increase the risk of irAEs by mediating irAE-related factors such as neutrophil and T-cell activation. In particular, use of antibiotics with anaerobic activity, given after initiation of PD-1/PD-L1 inhibitors, has been associated with an increased risk of more severe ICI-mediated diarrhea and/or colitis (IMDC) [21]. Because most of the normal gut flora in humans is anaerobic, and some of these beneficial anaerobes (such as *Akkermansia muciniphila*) reduce colitis, antibiotics that target anaerobes can disrupt the gut flora. Accordingly, adverse gut microbiota changes due to the use of antibiotics with anaerobic activity may lead to altered immune system regulation, thereby contributing to the development of IMDC. These findings suggest that in cancer patients treated with PD-1/PD-L1 inhibitors, the decision to administer antibiotics should be considered carefully to avoid increasing the risk of irAEs.

In the present study, we observed a longer OS in patients who did not develop irAEs than in those who did. In the subgroup analysis of the four most common irAEs, dermatologic- and thyroid-related irAEs did not significantly affect OS, but heart- and lung-related irAEs were significantly associated with poorer OS survival. However, the prediction of irAEs and prognosis of patients receiving immunotherapy has been controversial [22, 23]. A retrospective study conducted at Johns Hopkins Hospital showed the mortality risk among patients who developed pneumonia after receiving immunotherapy was 2.7 times that of patients who did not develop pneumonia [24], which is consistent with our findings. Notably, the occurrence of irAEs may be correlated with the efficacy and prognosis of immunotherapy, but it is not positively correlated. In particular, high-grade irAEs may directly lead to severe organ damage and even death. However, as a macroscopic manifestation of off-target treatment effects, the predictive relationship between irAEs and treatment efficacy has been gradually questioned. Predicting treatment efficacy based on irAEs ignores survivorship bias, as adequate survival is required for the observation of irAEs to be possible [25]. In addition, some patients with hyper-progression have an extremely poor prognosis and die early before the occurrence of irAEs. Inclusion of these patients in the non-irAEs group introduces bias, which will obviously greatly reduce the reliability of survival data for this group. Therefore, the use of irAE occurrence to predict treatment efficacy needs to be studied with more caution.

IrAEs are essentially inflammatory responses caused by excessive activation of the immune system. Steroids have powerful and rapid anti-inflammatory and anti-immune effects, making them the first-line treatment strategy for irAEs. Given the immunosuppressive effect of steroids, whether their use affects the efficacy of immunotherapy is worth exploring [26]. In the present study, some patients were given corticosteroids to prevent and reduce adverse reactions to combined chemotherapy drugs (such as nausea and vomiting, allergic reactions, etc.), and a lower dose of 10 mg dexamethasone was used. The results showed that use of low-dose corticosteroids had no significant effect on the survival of patients. The KEYNOTE-189 [27] and KEYNOTE-407 [28] large phase III RCT studies both found that short-term pretreatment with low-dose hormones in patients who received immunotherapy combined with chemotherapy did not significantly affect the efficacy of ICIs. Additionally, other studies have shown that immunotherapy efficacy was worse in patients receiving ≥ 10 mg prednisone than in those receiving 0–10 mg, and pointed out that differences in the efficacy of corticosteroid therapy may be due to specific factors related to the need for palliative steroid treatment [26, 29]. In conclusion, it is recommended that the indications for medium to high doses of hormone use should be carefully considered before ICI use.

Our investigation of predictors of the severity of irAEs revealed that patients with a BMI ≥ 25 kg/m^2^ at baseline had irAEs of a higher severity grade than those with a BMI < 25 kg/m^2^. The role of BMI in the occurrence and development of cancer and immunotherapy has been the focus of extensive research. The latest global burden of cancers study found that high BMI is one of the main factors leading to cancer-related death [30]. A real-world retrospective study of 1070 cancer patients revealed that obesity was the only factor significantly associated with an increased incidence of grade 3–4 irAEs [31]. Pollack et al. reported that baseline BMI is an independent predictor of thyroid-related irAEs and a higher baseline BMI corresponds to the earlier occurrence of significant thyrotoxicosis [32]. Furthermore, obesity appears to be associated with an increased incidence of grade 3–4 irAEs in women treated with Nivolumab [33]. Evidence of the relationship between BMI and response to immunotherapy demonstrates an “obesity paradox,” in which a higher BMI appears to be associated with better responses to ICI therapy [34]. However, when immunotherapy is combined with chemotherapy, this benefit of BMI is lost [35]. Mechanistically, obesity promotes a chronic inflammatory state that predisposes individuals to metabolic disorders and immune-mediated conditions [33]. Obesity leads to T-cell dysfunction and increases depletion of T cells with a PD-1-positive phenotype in the adipose tissue and TME through leptin production, whereas ICI therapy inhibits T cells by uncoupling PD-1/PD-L1 binding. Accordingly, obese patients with T-cell exhaustion may respond well to ICI therapy [36]. The mechanism by which ICI therapy acts to increase the responsiveness of a patient’s T cells may be affected when chemotherapy is added [35]. In addition, visceral adipocytes secrete various cytokines such as tumor necrosis factor alpha (TNF-α), IL-6, and IL-1β, leading to T helper cell 1 (Th1)/Th2 imbalance and promoting a proinflammatory state [33]. Recent studies have demonstrated that the prognostic effects of obesity and visceral adiposity on patient survival are dependent on the systemic immune-inflammatory state represented by a systemic immune-inflammatory index, suggesting that systemic inflammation may be the basis driving the obesity paradox [37]. Considering the associations between higher BMI and improved clinical outcomes, the finding of an association between high BMI and high-grade irAEs may be the basis of an “immunogenic phenotype” [32].

Among peripheral blood factors, a lower baseline PNI and higher post-treatment LDH level tended to develop irAEs with higher severity grades. Systemic inflammation and nutritional disorders promote cell carcinogenesis by inhibiting apoptosis, promoting angiogenesis, and damaging DNA. The PNI, an indicator of nutritional status and systemic immune capacity, has been shown to be an independent prognostic factor for a variety of cancers [38]. Previous studies have shown that PNI is significantly correlated with tumor infiltrating lymphocyte status, the density of CD4 + and CD8 + immune cells, and systemic inflammation [39, 40]. Malnutrition plays an important role in increasing treatment complications, diminishing quality of life, and increasing mortality among cancer patients. Collectively, patients with a lower PNI have poorer nutritional status, are unable to tolerate the systemic immune inflammatory response by immunotherapy, and are prone to more severe irAEs and worse prognosis. LDH, as a complex biomarker, is associated with the activation of a variety of oncogenic signaling pathways and the metabolic activity, aggressiveness, proinflammatory state, and immunogenicity of many tumors. High levels of LDH are associated with a higher tumor burden and poor prognosis after immunotherapy [41]. Additional studies support LDH as an important target in cancer therapy [42, 43]. A previous study demonstrated elevated LDH in myocarditis associated with immune checkpoint suppression [44]. Therefore, paying attention to the PNI and LDH levels as biomarkers can help clinicians identify more severe irAEs.

Although our study did not show a correlation between different types of ICI antibodies and the incidence of irAEs, a meta-analysis including 125 clinical studies of PD-1/PD-L1 monotherapy showed that the incidence of grade 3 and above irAEs was higher in patients treated with PD-1 inhibitors than in patients treated with PD-L1 inhibitors (OR, 1.58; 95% CI, 1.00–2.54), which may be related to the preservation of the PD-L2 pathway by PD-L1 inhibitors and maintenance of local homeostasis of PD-L2 [45, 46]. In addition, the cumulative effect of combination therapy has raised concerns about serious and even life-threatening irAEs. Another meta-analysis showed that chemotherapy and PD-L1 inhibitor combination therapies were associated with a higher risk of irAEs, and specifically a greater risk of grade 3 or higher irAEs, than targeted therapy combinations and immunotherapy combinations [47]. Other studies have shown that patients receiving combination therapy had a higher incidence of myocarditis than those receiving immune monotherapy [48]. Furthermore, in a study of sequential treatment with radiotherapy and immunotherapy, ICIs administration within 90 days after radiotherapy was not associated with an increased risk of severe AEs, and therefore, ICIs administration within 90 days after radiotherapy was considered safe [49]. However, no correlations were found between the risk of irAEs and different combination therapies in our study, which may be due to the fact that our study was a single-center, real-world retrospective study. However, this potential increase in side effects represents a great challenge for the development of novel combinations based on PD-1 and PD-L1 inhibitors, especially given the increased clinical use of combination treatments. Therefore, it is necessary to characterize the incidence and toxicity profiles of PD-1 and PD-L1 inhibitor-based combinations in a standardized way to guide clinicians in better assessing treatment risk and managing potential irAEs.

Combination of multiple detection methods may be the trend for irAE prediction in the future. Jing et al. investigated potential predictors of irAE risk in patients receiving anti-PD-1/PD-L1 therapy for 26 different tumor types by integrating real-world pharmacoalertness and molecular omics data and showed that a bivariate linear-regression model based on lymphocyte cystolic protein 1 and adenosine diphosphate-dependent glucokinase expression could accurately predict irAEs [50]. Multidirectional, comprehensive biomarkers are needed for the early identification of irAEs and subsequent therapeutic intervention. We expect that in future studies, ideal biomarkers that are accurate, reliable, economical, and convenient can be screened for dynamic monitoring before and during immunotherapy, and their practical clinical application value can be verified through large-scale, multi-center clinical trials.

Some limitations of the present study must be noted. First, our study cohort was from a single institution, which increases the risk of regional, site-specific, or physician treatment bias. Second, the variety of tumor types included in the design of this study resulted in heterogeneity within the sample. Finally, the short survival follow-up affected the stability of the survival analysis results. Therefore, multi-center, prospective studies are needed to validate the results of our study.

## Conclusions

The present study explored potential predictors of irAEs in patients who received PD-1/PD-L1 inhibitor treatment and identified female gender, antibiotic use, baseline CTC level, and post-treatment NLR as predictive biomarkers for the occurrence of irAEs. Further analysis of irAEs of differing severity levels revealed the predictive value of the PNI, BMI, and LDH levels. In addition, the use of low-dose steroids did not have a significant on patient survival in this study. Patients who did not experience irAEs had better OS than patients who did experience irAEs. Immune-related pneumonitis and cardiotoxicity were found to be significant predictors of poor prognosis in the subgroup analysis of patients with different organ-related irAEs.

### Electronic supplementary material

Below is the link to the electronic supplementary material.

Supplementary Material 1

## Data Availability

The datasets generated and/or analyzed during the current study are not publicly available due to personal privacy but are available from the corresponding author on reasonable request.

## References

[1] Wang J, Ma X, Ma Z, Ma Y, Wang J, Cao B (2022). Research Progress of biomarkers for Immune Checkpoint inhibitors on Digestive System Cancers. Front Immunol.

[2] Postow MA, Sidlow R, Hellmann MD (2018). Immune-related adverse events Associated with Immune Checkpoint Blockade. N Engl J Med.

[3] Ramos-Casals M, Brahmer JR, Callahan MK, Flores-Chávez A, Keegan N, Khamashta MA (2020). Immune-related adverse events of checkpoint inhibitors. Nat Reviews Disease Primers.

[4] Sullivan RJ, Weber JS (2022). Immune-related toxicities of checkpoint inhibitors: mechanisms and mitigation strategies. Nat Rev Drug Discov.

[5] Dougan M, Luoma AM, Dougan SK, Wucherpfennig KW (2021). Understanding and treating the inflammatory adverse events of cancer immunotherapy. Cell.

[6] Finlay WJJ, Coleman JE, Edwards JS, Johnson KS (2019). Anti-PD1 ‘SHR-1210’ aberrantly targets pro-angiogenic receptors and this polyspecificity can be ablated by paratope refinement. MAbs.

[7] Kennedy LB, Salama AKS (2020). A review of cancer immunotherapy toxicity. CA Cancer J Clin.

[8] Han J, Yang Y, Li X, Wu J, Sheng Y, Qiu J (2022). Pan-cancer analysis reveals sex-specific signatures in the tumor microenvironment. Mol Oncol.

[9] Pala L, De Pas T, Catania C, Giaccone G, Mantovani A, Minucci S (2022). Sex and cancer immunotherapy: current understanding and challenges. Cancer Cell.

[10] Unger JM, Vaidya R, Albain KS, LeBlanc M, Minasian LM, Gotay CC (2022). Sex differences in risk of severe adverse events in patients receiving immunotherapy, targeted therapy, or Chemotherapy in Cancer clinical trials. J Clin Oncology: Official J Am Soc Clin Oncol.

[11] Park JW, Chang HJ, Yeo HY, Han N, Kim BC, Kong SY (2020). The relationships between systemic cytokine profiles and inflammatory markers in colorectal cancer and the prognostic significance of these parameters. Br J Cancer.

[12] Valero C, Lee M, Hoen D, Weiss K, Kelly DW, Adusumilli PS (2021). Pretreatment neutrophil-to-lymphocyte ratio and mutational burden as biomarkers of tumor response to immune checkpoint inhibitors. Nat Commun.

[13] Daniello L, Elshiaty M, Bozorgmehr F, Kuon J, Kazdal D, Schindler H (2021). Therapeutic and prognostic implications of Immune-related adverse events in Advanced Non-small-cell Lung Cancer. Front Oncol.

[14] Aceto N, Bardia A, Miyamoto DT, Donaldson MC, Wittner BS, Spencer JA (2014). Circulating tumor cell clusters are oligoclonal precursors of breast cancer metastasis. Cell.

[15] Tamminga M, de Wit S, Hiltermann TJN, Timens W, Schuuring E, Terstappen LW (2019). Circulating tumor cells in advanced non-small cell lung cancer patients are associated with worse tumor response to checkpoint inhibitors. J Immunother Cancer.

[16] Castello A, Carbone FG, Rossi S, Monterisi S, Federico D, Toschi L (2020). Circulating Tumor cells and metabolic parameters in NSCLC patients treated with checkpoint inhibitors. Cancers (Basel).

[17] Bomze D, Hasan Ali O, Bate A, Flatz L (2019). Association between Immune-related adverse events during Anti-PD-1 therapy and Tumor Mutational Burden. JAMA Oncol.

[18] Indini A, Rijavec E, Grossi F (2021). Circulating biomarkers of response and toxicity of Immunotherapy in Advanced Non-small Cell Lung Cancer (NSCLC): a Comprehensive Review. Cancers (Basel).

[19] Mohiuddin JJ, Chu B, Facciabene A, Poirier K, Wang X, Doucette A (2021). Association of antibiotic exposure with survival and toxicity in patients with Melanoma receiving immunotherapy. J Natl Cancer Inst.

[20] Jing Y, Chen X, Li K, Liu Y, Zhang Z, Chen Y (2022). Association of antibiotic treatment with immune-related adverse events in patients with cancer receiving immunotherapy. J Immunother Cancer.

[21] Abu-­Sbeih H, Herrera LN, Tang T (2019). Impact of antibiotic therapy on the development and response to treatment of immune checkpoint inhibitor-mediated diarrhea and colitis. J Immunother Cancer.

[22] Shankar B, Zhang J, Naqash AR, Forde PM, Feliciano JL, Marrone KA (2020). Multisystem Immune-related adverse events Associated with Immune Checkpoint inhibitors for treatment of Non-small Cell Lung Cancer. JAMA Oncol.

[23] Haratani K, Hayashi H, Chiba Y, Kudo K, Yonesaka K, Kato R (2018). Association of Immune-related adverse events with Nivolumab Efficacy in Non-small-cell Lung Cancer. JAMA Oncol.

[24] Suresh K, Psoter KJ, Voong KR, Shankar B, Forde PM, Ettinger DS (2019). Impact of checkpoint inhibitor pneumonitis on Survival in NSCLC patients receiving Immune Checkpoint Immunotherapy. J Thorac Oncol.

[25] Zhang B, Nie W, Han B (2021). Immune-related adverse events and efficacy-the more it hurts, the Better It Works?. JAMA Oncol.

[26] Arbour KC, Mezquita L, Long N, Rizvi H, Auclin E, Ni A (2018). Impact of baseline steroids on efficacy of programmed cell Death-1 and programmed death-ligand 1 blockade in patients with non-small-cell Lung Cancer. J Clin Oncol.

[27] Gandhi L, Rodríguez-Abreu D, Gadgeel S, Esteban E, Felip E, De Angelis F (2018). Pembrolizumab plus Chemotherapy in Metastatic Non-small-cell Lung Cancer. N Engl J Med.

[28] Paz-Ares L, Luft A, Vicente D, Tafreshi A, Gümüş M, Mazières J (2018). Pembrolizumab plus Chemotherapy for squamous non-small-cell Lung Cancer. N Engl J Med.

[29] Ricciuti B, Dahlberg SE, Adeni A, Sholl LM, Nishino M, Awad MM (2019). Immune checkpoint inhibitor outcomes for patients with non-small-cell Lung Cancer receiving baseline corticosteroids for Palliative Versus Nonpalliative indications. J Clin Oncol.

[30] GBD 2019 Cancer Risk Factors Collaborators (2022). The global burden of cancer attributable to risk factors, 2010-19: a systematic analysis for the global burden of Disease Study 2019. Lancet.

[31] Cortellini A, Bersanelli M, Santini D, Buti S, Tiseo M, Cannita K (2020). Another side of the association between body mass index (BMI) and clinical outcomes of cancer patients receiving programmed cell death protein-1 (PD-1)/ programmed cell death-ligand 1 (PD-L1) checkpoint inhibitors: a multicentre analysis of immune-related adverse events. Eur J Cancer.

[32] Pollack R, Ashash A, Cahn A, Rottenberg Y, Stern H, Dresner-Pollak R (2020). Immune Checkpoint inhibitor-induced thyroid dysfunction is Associated with higher body Mass Index. J Clin Endocrinol Metab.

[33] McQuade JL, Hammers H, Furberg H, Engert A, André T, Blumenschein G, et al. Association of Body Mass Index with the Safety Profile of Nivolumab with or without Ipilimumab. JAMA Oncol. 2022;5409. 10.1001/jamaoncol.2022.5409. 10.1001/jamaoncol.2022.5409 PMC9857666 36480191

[34] Kichenadasse G, Miners JO, Mangoni AA, Rowland A, Hopkins AM, Sorich MJ (2020). Association between Body Mass Index and overall survival with Immune checkpoint inhibitor therapy for Advanced Non-small Cell Lung Cancer. JAMA Oncol.

[35] Cortellini A, Ricciuti B, Vaz VR, Soldato D, Alessi JV, Dall’Olio FG (2022). Prognostic effect of body mass index in patients with advanced NSCLC treated with chemoimmunotherapy combinations. J Immunother Cancer.

[36] Wang Z, Aguilar EG, Luna JI, Dunai C, Khuat LT, Le CT (2019). Paradoxical effects of obesity on T cell function during tumor progression and PD-1 checkpoint blockade. Nat Med.

[37] Lee JH, Hyung S, Lee J, Choi SH (2022). Visceral adiposity and systemic inflammation in the obesity paradox in patients with unresectable or metastatic melanoma undergoing immune checkpoint inhibitor therapy: a retrospective cohort study. J Immunother Cancer.

[38] Qu Z, Lu Y-J, Feng J-W, Chen YX, Shi LQ, Chen J (2021). Preoperative Prognostic Nutritional Index and Neutrophil-to-lymphocyte ratio Predict Survival outcomes of patients with Hepatocellular Carcinoma after curative resection. Front Oncol.

[39] Wang JB, Li P, Liu XL, Zheng QL, Ma YB, Zhao YJ (2020). An immune checkpoint score system for prognostic evaluation and adjuvant chemotherapy selection in gastric cancer. Nat Commun.

[40] Okadome K, Baba Y, Yagi T, Kiyozumi Y, Ishimoto T, Iwatsuki M (2020). Prognostic Nutritional Index, Tumor-infiltrating lymphocytes, and prognosis in patients with esophageal Cancer. Ann Surg.

[41] Rosner S, Kwong E, Shoushtari AN, Friedman CF, Betof AS, Brady MS (2018). Peripheral blood clinical laboratory variables associated with outcomes following combination nivolumab and ipilimumab immunotherapy in melanoma. Cancer Med.

[42] Certo M, Tsai CH, Pucino V, Ho PC, Mauro C (2021). Lactate modulation of immune responses in inflammatory versus tumour microenvironments. Nat Rev Immunol.

[43] Claps G, Faouzi S, Quidville V, Chehade F, Shen S, Vagner S (2022). The multiple roles of LDH in cancer. Nat Rev Clin Oncol.

[44] Xie X, Wang L, Li Y, Xu Y, Wu J, Lin X (2022). Multi-organ Immune-related adverse event is a risk factor of Immune Checkpoint inhibitor-Associated myocarditis in Cancer patients: a multi-center study. Front Immunol.

[45] Yu J, Song Y, Tian W (2020). How to select IgG subclasses in developing anti-tumor therapeutic antibodies. J Hematol Oncol.

[46] Wang Y, Zhou S, Yang F, Qi X, Wang X, Guan X (2019). Treatment-related adverse events of PD-1 and PD-L1 inhibitors in clinical trials: a systematic review and Meta-analysis. JAMA Oncol.

[47] Zhou X, Yao Z, Bai H, Duan J, Wang Z, Wang X (2021). Treatment-related adverse events of PD-1 and PD-L1 inhibitor-based combination therapies in clinical trials: a systematic review and meta-analysis. Lancet Oncol.

[48] Naqash AR, Moey MYY, Cherie Tan XW, Laharwal M, Hill V, Moka N (2022). Major adverse cardiac events with Immune Checkpoint inhibitors: a pooled analysis of trials Sponsored by the National Cancer Institute-Cancer therapy evaluation program. J Clin Oncol.

[49] Anscher MS, Arora S, Weinstock C, Amatya A, Bandaru P, Tang C (2022). Association of Radiation Therapy with risk of adverse events in patients receiving immunotherapy: a pooled analysis of trials in the US Food and Drug Administration Database. JAMA Oncol.

[50] Jing Y, Liu J, Ye Y, Pan L, Deng H, Wang Y (2020). Multi-omics prediction of immune-related adverse events during checkpoint immunotherapy. Nat Commun.

