# Supplementary figures and images for: Predictive biomarkers for immune-related adverse events in cancer patients treated with immune-checkpoint inhibitors
Source: BMC Immunol. 2024 Jan 24;25:8. doi: 10.1186/s12865-024-00599-y (PMC10809515; doi:10.1186/s12865-024-00599-y)

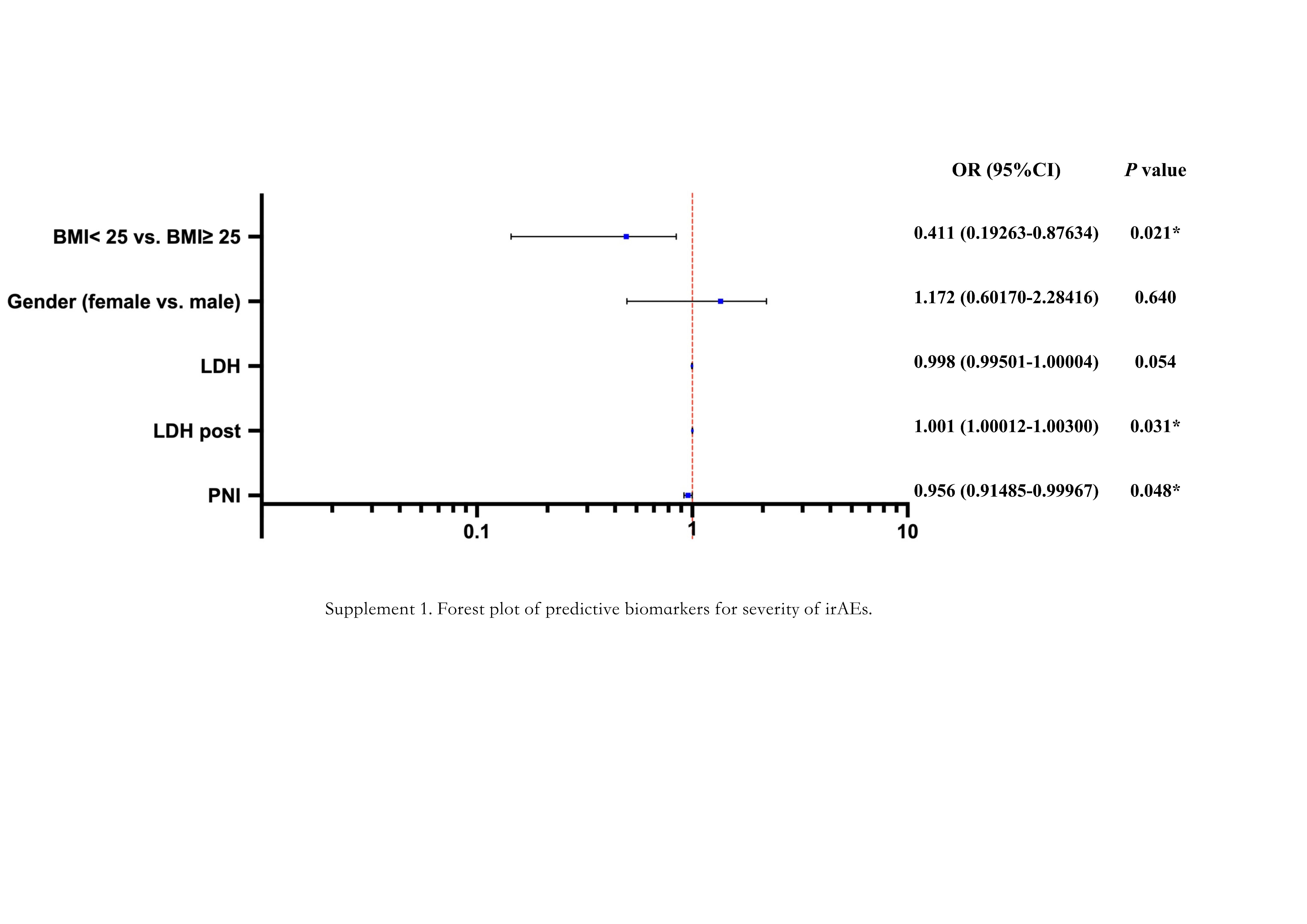

Supplement: Supplementary file 1 — Supplementary Material 1 [file 12865_2024_599_MOESM1_ESM.jpg]
